# Peer review of "Metabolite Profiling and Association Analysis of Leaf Tipburn in Heat-Tolerant Bunching Onion Varieties"

_plants, 2025, doi:10.3390/plants14020187_

Round 1

Reviewer 1 Report

Comments and Suggestions for Authors

The paper by Nakajima et al., entitled Metabolite Profiling of Heat-Tolerant Bunching Onion Varieties Possessing Dark Green Leaf Blade Coloration and Its Application to Association Analyses For Leaf Tipburnis timely, addressing the impact of climate change on crop quality, which is a pressing issue in agriculture today. The study is scientifically sound, employing metabolomics to explore a significant agricultural problem. However, the methodology and results need clearer articulation to support this soundness. Authors are suggested with some revisions before formal acceptance in the journal. Find the comments below;

l  The title is informative but could be more concise. Consider simplifying it to enhance clarity while retaining key terms like "metabolite profiling" and "leaf tipburn".

l  The abstract lacks specific quantitative results. Including key findings, such as the percentage of metabolites identified or specific compounds linked to leaf tipburn, would strengthen it.

l  The introduction provides a good background but could benefit from a clearer statement of the research gap. Explicitly stating what previous studies have missed would enhance its relevance.

l  The methodology section should include more details on the sample size and selection criteria. This information is crucial for reproducibility and understanding the study's scope.

l  Statistical methods are mentioned, a more detailed explanation of the PLS-DA and its relevance to the study's objectives would improve clarity. Including assumptions and limitations of the methods used is also essential.

l  The results section could be improved by providing more context for the findings. For instance, explaining the significance of the identified metabolites in relation to leaf tipburn would enhance understanding.

l  The figures and tables should be more clearly labeled and referenced in the text. Providing legends that explain the significance of each figure would help readers interpret the data better.

l  The discussion should delve deeper into the implications of the findings. Discussing how the identified metabolites can be utilized in practical applications or future research would add value.

l  The conclusion should summarize the key findings more effectively and suggest specific future research directions. This would provide a clearer pathway for subsequent studies.

l  The paper lacks citations for some claims made in the introduction and discussion. Ensuring all statements are backed by relevant literature is crucial for scientific rigor.

l  The analysis of organosulfur compounds is intriguing but could be expanded. Discussing their roles in stress response mechanisms would provide a more comprehensive understanding.

l  The paper mentions environmental factors influencing leaf tipburn but could benefit from a more detailed exploration of how these factors interact with the identified metabolites.

Comments on the Quality of English Language

ok

Author Response

Thank you very much for taking the time to review this manuscript. Please find the detailed responses below, with the corresponding revisions/corrections marked in red within the text of the resubmitted files.

Comments 1:  The title is informative but could be more concise. Consider simplifying it to enhance clarity while retaining key terms like "metabolite profiling" and "leaf tipburn".

Response 1: Thank you for your feedback. We have focused on key points and made the title more concise.

[Metabolite Profiling and Association Analysis of Leaf Tipburn in Heat-Tolerant Bunching Onion Varieties]

Comments 2: The abstract lacks specific quantitative results. Including key findings, such as the percentage of metabolites identified or specific compounds linked to leaf tipburn, would strengthen it.

Response 2: Thank you for your feedback. We inserted the following sentence.

(Line 16-18) Based on the intensity data, the fold change of this metabolite was calculated to be 1.66, indicating an increase in the leaf tipburn group compared to the control group.

Comments 3:   The introduction provides a good background but could benefit from a clearer statement of the research gap. Explicitly stating what previous studies have missed would enhance its relevance.

Response 3: Thank you for your feedback. We inserted the following sentence at line 68-76.

Despite extensive research on tipburn in leafy crops such as lettuce (Lactuca sativa L. ), onion, and cauliflower (Brassica oleracea L.), most of these studies have primarily focused on nutritional deficiencies or genotype- related tipburn symptoms [ 18, 28, 29 ]. There is limited knowledge of the biochemical and physiological mechanisms underlying this disorder in bunching onions, particularly under combined environmental stresses. This study addresses this gap through integrating metabolomics with pigment and functional component analyses to uncover the mechanisms of tipburn development and identify strategies for improving tolerance in heat-tolerant F1 hybrid and purebred varieties.

Comments 4: The methodology section should include more details on the sample size and selection criteria. This information is crucial for reproducibility and understanding the study's scope.

Response 4: Thank you for your feedback. We inserted the following sentence at line 91-95.

This experiment aimed to screen for the conditions and cultivars most susceptible to leaf tipburn. To make the most of the limited greenhouse space and resources, we selected nine cultivars with three replications and six growth conditions to reflect diverse genetic and environmental scenarios.

Comments 5: Statistical methods are mentioned, a more detailed explanation of the PLS-DA and its relevance to the study's objectives would improve clarity. Including assumptions and limitations of the methods used is also essential.

Response 5: Thank you for your feedback. We inserted the following sentence.

(Line258-261) This method is particularly suitable for high-dimensional metabolomic data as it emphasizes group separation and identifies key metabolites using VIP scores. However, despite limitations such as assumptions of linearity and the risk of overfitting, it remains a valuable tool for exploring group separation in high-dimensional datasets.

(Line 272-274) This method was chosen to address the limitations of PLS-DA, specifically its inability to capture non-linear relationships, and to mitigate the risk of overfitting inherent in single decision tree models.

Comments 6:  The results section could be improved by providing more context for the findings. For instance, explaining the significance of the identified metabolites in relation to leaf tipburn would enhance understanding.

Response 6: Thank you for your feedback. We inserted the following sentence at line 368-373.

Gamma-Glu-PRENCSO is an intermediate of PRENCSO, a precursor of pungency-related compounds. In the leaf tipburn group, organosulfur compounds tended to proceed toward the alliin synthesis pathway rather than the sulfur- assimilation- or antioxidant-related pathways. This metabolic shift may lead to a deficiency in metabolites essential for stress responses, potentially resulting in inadequate adaptation to stress in the leaf tipburn group.

Comments 7:  The figures and tables should be more clearly labeled and referenced in the text. Providing legends that explain the significance of each figure would help readers interpret the data better.

Response 7: Thank you for your feedback. We have revised the captions for each figure and table, except for Figure 5.

Comments 8: The discussion should delve deeper into the implications of the findings. Discussing how the identified metabolites can be utilized in practical applications or future research would add value.

Response 8: Thank you for your feedback. We inserted the following sentence at line 474-488.

These results suggest that enhancing flavonoid accumulation and activating GGT to reduce gamma-Glu-PRENCSO could be critical strategies for mitigating oxidative stress and suppressing leaf tipburn. Our findings highlight the dual role of flavonoids as antioxidants and signaling molecules that can modulate plant responses to environmental stress and leaf tipburn symptoms, providing a biochemical foundation for developing stress-tolerant varieties. The activation of GGT to decrease gamma-Glu-PRENCSO levels could also serve as a metabolic intervention to reduce the buildup of reactive oxygen species, thereby preventing cellular damage and suppressing leaf tipburn. This insight could be applied in practical breeding programs to develop stress-resistant cultivars by targeting metabolic pathways associated with sulfur metabolism and flavonoid biosynthesis. Furthermore, future studies could explore the feasibility of using gamma-Glu-PRENCSO and flavonoids as biomarkers to monitor stress levels and predict leaf tipburn incidence in agricultural practices. Additionally, future research could explore the genetic and environmental factors influencing these metabolic pathways, as well as their interactions with other stress response mechanisms, to develop comprehensive strategies for improving crop resilience.

Comments 9: The conclusion should summarize the key findings more effectively and suggest specific future research directions. This would provide a clearer pathway for subsequent studies.

Response 9: Thank you for your feedback. We inserted the following sentence at line 490-502.

In this study, metabolome profiling was conducted to compare leaf tipburn and control groups, revealing that organic sulfur compounds are involved in leaf tipburn. Among these, gamma-Glu-PRENCSO was found to significantly increase in the leaf tipburn group. Gamma-Glu-PRENCSO is an intermediate of PRENCSO, a precursor of pungency, and is synthesized when GGT removes the gamma-glutamyl group. This gamma-glutamyl group functions as a substrate for glutathione, which plays a crucial role in stress responses, reactive oxygen species scavenging, and the recycling of sulfur compounds. These findings suggest that activating GGT to reduce gamma-Glu-PRENCSO accumulation could be a promising strategy to enhance stress tolerance and mitigate leaf tipburn. Future research will aim to monitor the dynamics of gamma-Glu-PRENCSO under various conditions to further elucidate the mechanisms underlying leaf tipburn. Additionally, based on these insights, efforts will be made to establish optimal cultivation conditions and develop stress-tolerant cultivars.

Comments 10: The paper lacks citations for some claims made in the introduction and discussion. Ensuring all statements are backed by relevant literature is crucial for scientific rigor.

Response 10: Thank you for your feedback. We have added the citations as follows.

Introduction: Line31, 45, and 71
Discusssion:Line416, 439,, 445, 452, 455, and 456

Comments 11: The analysis of organosulfur compounds is intriguing but could be expanded. Discussing their roles in stress response mechanisms would provide a more comprehensive understanding.

Response 11: Thank you for your valuable feedback. We have incorporated additional information and made revisions regarding stress responses in Line 441-459

Organosulfur compounds are involved in various stress responses, including the scavenging of reactive oxygen species (ROS) by glutathione, the synthesis of ethylene from methionine, and signal transduction via hydrogen sulfide (H2S). Notably, H2S plays a key role in inducing stomatal closure, promoting nitric oxide production, and enhancing ABA synthesis [ 44 –46]. Comparative analyses of organosulfur compounds between the control and leaf tipburn groups revealed significant differences in key metabolic pathways. In the control group, metabolites associated with sulfur assimilation, glutathione, and methionine biosynthesis were increased, while gamma-Glu-PRENCSO, a precursor of pungency, was decreased (Figrure 8). Gamma-Glu-PRENCSO is converted into PRENCSO, a precursor of pungency, by the action of gamma-glutamyl transferase (GGT), which cleaves the gamma-Glu group [47]. GGT plays a critical role in the synthesis of glutathione, which is involved in scavenging reactive oxygen species, by transferring gamma-Glu groups to specific amino acids or peptides. In Arabidopsis, GGT has been reported to be essential for mitigating oxidative stress [ 48]. Oxidative stress is caused by various factors, including environmental (abiotic) stress [ 49], and the ability to appropriately respond to such stress has been suggested to influence the occurrence of leaf tipburn. These findings indicate that stress-tolerant metabolic processes were more active in the control group, whereas these processes were diminished in the leaf tipburn group.

Comments 12: The paper mentions environmental factors influencing leaf tipburn but could benefit from a more detailed exploration of how these factors interact with the identified metabolites.

Response 12: Thank you for your valuable feedback. We have added details about the interaction of flavonoids and organosulfur compounds with environmental factors identified in this study as follows.

(Line438-441) Flavonoids are one of the defensive antioxidant substances that play a critical role in plant stress responses [43]. They contribute to alleviating oxidative stress and protecting cell membranes, potentially influencing the occurrence of leaf tipburn through metabolic changes under stress conditions.

(Line445-458) Comparative analyses of organosulfur compounds between the control and leaf tipburn groups revealed significant differences in key metabolic pathways. In the control group, metabolites associated with sulfur assimilation, glutathione, and methionine biosynthesis were increased, while gamma-Glu-PRENCSO, a precursor of pungency, was decreased (Figrure 8). gamma-Glu-PRENCSO is converted into PRECSO, a precursor of pungency, by the action of gamma-glutamyl transferase (GGT), which cleaves the gamma-Glu group [47]. GGT plays a critical role in the synthesis of glutathione, which is involved in scavenging reactive oxygen species, by transferring gamma-Glu groups to specific amino acids or peptides. In Arabidopsis, GGT has been reported to be essential for mitigating oxidative stress [48]. Oxidative stress is caused by various factors, including environmental (abiotic) stress [49], and the ability to appropriately respond to such stress has been suggested to influence the occurrence of leaf tipburn. These findings indicate that stress-tolerant metabolic processes were more active in the control group, whereas these processes were diminished in the leaf tipburn group.

Reviewer 2 Report

Comments and Suggestions for Authors

The study of Tetsuya Nakajima and co-authors entitled ” Metabolite Profiling of Heat-Tolerant Bunching Onion Varieties Possessing Dark Green Leaf Blade Coloration and Its Application to Association Analyses For Leaf Tipburn” is relevant, well-designed and performed using an adequate and modern methods. The obtained results are well-presented and discussed. The authors have done a lot of work and obtained important results. A widely targeted metabolomic analysis was performed to identify metabolites potentially involved in the development of leaf tipburn, offering a deeper understanding of the physiological responses linked to this disorder. The results emphasize the importance of sulfur metabolism and hormone regulation in stress tolerance and provide insights into potential mechanisms contributing to onion leaf tip burn.

The paper may be accepted for publication after double-checking the text.

Recommendations: Provide references to the literature for the methods used (Lines 119-123, 124-149, 150-157).

Line 359. Change ‘along with abscisic acid (ABA) and jasmonic acid (JA)’ to ‘along with ABA and JA’.  Abbreviations should be defined the first time they appear in each of three sections: the abstract, the main text, the first figure or table. Check, please. 

Also, the additional sections must be added before References: Author Contributions, Funding, Conflicts of Interest, etc. (https://www.mdpi.com/journal/plants/instructions)

Best regards,

Reviewer

Author Response

Thank you very much for taking the time to review this manuscript. Please find the detailed responses below, with the corresponding revisions/corrections marked in red within the text of the resubmitted files.

Comments 1: Recommendations: Provide references to the literature for the methods used (Lines 119-123, 124-149, 150-157).

Response 1: Thank you for your feedback. We have added the citation for the method in Line 135, 139-140, and 166.

Comments 2: Change ‘along with abscisic acid (ABA) and jasmonic acid (JA)’ to ‘along with ABA and JA’.  Abbreviations should be defined the first time they appear in each of three sections: the abstract, the main text, the first figure or table. Check, please. 

Response 2: Thank you for your feedback. We appreciate your careful review of our manuscript. We have made the correction in Line 386.

Comments 3: Also, the additional sections must be added before References: Author Contributions, Funding, Conflicts of Interest, etc. (https://www.mdpi.com/journal/plants/instructions)

Response 3: Thank you for pointing out the inaccuracies. Although it was registered online, it was missing in the main text. We have added that information at Line507-517.

Author Contributions: Conceptualization, S.M.; Methodology, S.H., M.S., and M.Y.H.; Software, T.N. and M.F.; Validation, M.S. and M.Y.H.; Investigation, R.Y., K.M., M.S., and M.Y.H.; Resources, K.F.; Writing—original draft preparation, T.N.; Writing—review and editing, M.A. and S.M.; Visualization, T.N.; Project administration, S.M. All authors have read and agreed to the published version of the manuscript.

Funding: This research was funded by JSPS KAKENHI, grant number 21H02188. and by JST SPRING, Grant Number JPMJSP2111.

Institutional Review Board Statement: Not applicable.

Informed Consent Statement: Not applicable.

Conflicts of Interest: The authors declare no conflicts of interest.  

Reviewer 3 Report

Comments and Suggestions for Authors

As a reviewer for this article,here are several review comments I have provided to the authors to help the article further enhance its quality and impact:

• Lines 2-4:The background of global change may attract readers.

• Keywords should be arranged in order.

• The introduction should be divided into several paragraphs for easy understanding by readers,for example,the first segmentation at Line 33,and subsequent segmentations should be done by the authors themselves.

• The article mentions the impact of global warming on onion production and the issue of leaf tip necrosis in the introduction,but fails to fully elaborate on why heat-resistant and dark green leafed tillering onion varieties were chosen as research objects.It is recommended that the authors add this part to clarify the research motivation and the particularity of the selected varieties,so that readers can better understand the necessity and importance of the research.

• For the specific steps of metabolite extraction and analysis,some technical details can be added,such as the specific components of the extraction solvent used,extraction temperature,and time,etc.

• In-depth discussion of experimental results:

In the discussion section,the authors mention the importance of sulfur metabolism and hormone regulation in stress tolerance,but fail to fully elaborate on the practical guidance of these findings for onion cultivation and breeding.It is recommended that the authors,in conjunction with existing literature,delve into how these findings can provide new ideas and methods for improving the tolerance of onion varieties to leaf tip necrosis.

• Additionally,for metabolites with high VIP scores mentioned in the article,it is recommended that the authors further analyze their possible biological functions and regulatory mechanisms to provide new directions for future research.

• The overall structure of the article is clear,but there are logical jumps or repetitions between some paragraphs.It is recommended that the authors carefully comb through the structure of the article to ensure the logical coherence between paragraphs.

• In terms of language expression,it is recommended that the authors pay attention to the accuracy and conciseness of word usage,avoiding overly complex sentence structures or professional jargon,to facilitate reader understanding and acceptance.

In summary,through efforts such as clarifying the research background and motivation,detailing experimental materials and methods,strengthening data analysis and interpretation,in-depth discussion of experimental results,optimizing article structure and language expression,and supplementing references,this article can further enhance its academic value and impact.It is hoped that the authors will seriously consider these suggestions and make corresponding revisions and improvements to the article.

Comments on the Quality of English Language

need language improvement

Author Response

Thank you very much for taking the time to review our manuscript, despite the challenging circumstances. We understand that the review process can be time-consuming, and we truly appreciate the effort you put into assessing our work. Due to the delay in receiving feedback, an additional reviewer was introduced to expedite the process. However, your insights and considerations remain highly valued, and we sincerely thank you for your contribution to improving the quality of our manuscript.

Reviewer 4 Report

Comments and Suggestions for Authors

The manuscript (MP) titled " Metabolite Profiling of Heat-Tolerant Bunching Onion Varieties Possessing Dark Green Leaf Blade Coloration and Its Application to Association Analyses For Leaf Tipburn" submitted by Nakajima et al., investigated onion different varieties metabolite profiling during months and its application to association analyses for leaf tipburn. And I think it has certain application value for improving leaf tipburn tolerance and optimizing onion cultivation under challenging environmental conditions.

There are some concerns in this manuscript:

1, The title mentions heat-tolerance varieties, but the whole MP does not reflect that these onion varieties are heat-tolerance. The introduction also does not introduce relevant varieties, let alone provide references or data to prove that these varieties are indeed heat- tolerance.

2, In material methods, the data provided by the author for controlling the planting environment is not precise enough, and the author completely ignores the data such as light intensity, temperature fluctuations, and air humidity. These factors may affect the metabolism of scallions, thereby interfering with the accurate determination of metabolites related to leaf tip withering.

3,The authors used 9 different onion varieties. Has the impact of genetic background differences on metabolism been considered? How can the differences in genetic background between varieties affect their metabolic pathways and resistance mechanisms to leaf tip withering? It is suggested  that the author will provide more in-depth analysis and discussion in the article.

4, In terms of data analysis, although the multivariate statistical analysis methods such as PCA and PLS-DA used by the authors can reveal the overall trend and inter group differences of the data, their analytical ability for nonlinear relationships and interactions in complex metabolic data is limited, and some important metabolite information may have been overlooked.

5, The authors lack further validation experiments for the key metabolites and model prediction results related to leaf tip wilting identified through data analysis. For example, observing the changes in leaf tip withering symptoms of scallion after adding or inhibiting these metabolites in vitro.

Author Response

Thank you very much for taking the time to review this manuscript. Please find the detailed responses below, with the corresponding revisions/corrections marked in red within the text of the resubmitted files.

Comments 1: The title mentions heat-tolerance varieties, but the whole MP does not reflect that these onion varieties are heat-tolerance. The introduction also does not introduce relevant varieties, let alone provide references or data to prove that these varieties are indeed heat- tolerance.

Response 1: Thank you for your valuable feedback. The varieties used in this study include heat-tolerant varieties sold by seed companies and those bred by Yamaguchi Prefecture. To address the heat tolerance of bunching onions, we inserted the following sentence.

(Line33-35) In recent years, producers have sought traits such as "heat tolerance" to minimize leaf tipburn and poor growth during summer, as well as "dark green coloration" [1,4], and Japanese seed companies have developed summer F1 lines characterized by these traits.

(Line 79-80) from Nakahara Seed Product Co.,Ltd.

Comments 2: In material methods, the data provided by the author for controlling the planting environment is not precise enough, and the author completely ignores the data such as light intensity, temperature fluctuations, and air humidity. These factors may affect the metabolism of scallions, thereby interfering with the accurate determination of metabolites related to leaf tip withering.

Response 2: Thank you for your valuable feedback. We have obtained meteorological data, including the climatic conditions of Yamaguchi City and the solar radiation data from Fukuoka Prefecture, which is located near Yamaguchi City, from the Japan Meteorological Agency and added the following text to Lines 91–95.

This experiment aimed to screen for the conditions and cultivars most susceptible to leaf tipburn. To make the most of the limited greenhouse space and resources, we selected nine cultivars with three replications and six growth conditions to reflect diverse genetic and environmental scenarios.

Comments 3: The authors used 9 different onion varieties. Has the impact of genetic background differences on metabolism been considered? How can the differences in genetic background between varieties affect their metabolic pathways and resistance mechanisms to leaf tip withering? It is suggested  that the author will provide more in-depth analysis and discussion in the article.

Response 3: Thank you for your insightful feedback. We acknowledge the importance of considering the impact of genetic background differences on metabolism and resistance mechanisms to leaf tipburn. While our study primarily focused on metabolic differences related to leaf tipburn severity, we observed during clustering analysis that the metabolite profiles did not clearly segregate the varieties into distinct genetic groups, including fixed varieties, existing F1 varieties, and Yamaguchi F1 varieties. This observation led us to prioritize analyses on individuals with high and low leaf tipburn rates, as these distinctions were more pronounced in our dataset.

We agree that genetic background likely plays a role in shaping metabolic pathways and stress resistance mechanisms. However, our findings suggest that other factors, such as environmental conditions or complex metabolic interactions, may exert a stronger influence under the specific conditions of this study. Moving forward, we aim to integrate genomic and transcriptomic analyses into our research framework to better elucidate the contributions of genetic background to metabolic traits and stress responses. This approach will help bridge the gap between genotype and phenotype, providing deeper insights into the mechanisms underlying stress tolerance in bunching onions.

Comments 4: In terms of data analysis, although the multivariate statistical analysis methods such as PCA and PLS-DA used by the authors can reveal the overall trend and inter group differences of the data, their analytical ability for nonlinear relationships and interactions in complex metabolic data is limited, and some important metabolite information may have been overlooked.

Response 4: Thank you for pointing out the limitations of PCA and PLS-DA in analyzing non-linear relationships and interactions within complex metabolomic data. To address these limitations, we incorporated random forest analysis as an additional approach in this study. Random forest is a robust analytical method that considers non-linear relationships and prevents overfitting. However, we acknowledge that the context and details of this method were not sufficiently explained in the manuscript. Therefore, we have added the following text below to clarify our analytical approach.

(Line258-261) This method is particularly suitable for high-dimensional metabolomic data as it emphasizes group separation and identifies key metabolites using VIP scores. However, despite limitations such as assumptions of linearity and the risk of overfitting, it remains a valuable tool for exploring group separation in high-dimensional datasets.

(Line 272-274) This method was chosen to address the limitations of PLS-DA, specifically its inability to capture non-linear relationships, and to mitigate the risk of overfitting inherent in single decision tree models.

Comments 5: The authors lack further validation experiments for the key metabolites and model prediction results related to leaf tip wilting identified through data analysis. For example, observing the changes in leaf tip withering symptoms of scallion after adding or inhibiting these metabolites in vitro.

Response 5: Thank you for highlighting the need for further validation experiments to confirm the key metabolites associated with leaf tipburn and the model prediction results. We fully acknowledge the importance of experimental validation to strengthen our findings. However, it is important to note that genetic modification and genome editing techniques, such as CRISPR-Cas9, have not yet been fully established or widely applied to bunching onions. This technical limitation restricts our ability to directly manipulate the identified metabolites in this crop.

To address this challenge, our current approach is to identify or develop varieties in which the target compounds are naturally elevated. These naturally occurring variations will serve as a practical model for validating the role of the identified metabolites in leaf tipburn resistance. Additionally, we are exploring the possibility of leveraging similar crops within the Allium genus, such as bulb onions (A. cepa) or garlic (A. sativum), where more advanced molecular tools and protocols are available. These crops could provide alternative systems for functional validation, allowing us to extrapolate findings to bunching onions under comparable physiological and environmental conditions.

Furthermore, we aim to conduct complementary in vitro experiments, such as applying the metabolites exogenously to leaf tissues of similar crops or model plants, to observe their direct effects on tipburn symptoms. This stepwise approach not only accounts for the current technical limitations but also broadens the applicability of our findings across related species. We are confident that these strategies will contribute to a deeper understanding of the role of key metabolites in leaf tipburn and support the development of effective breeding or agronomic solutions for improving stress tolerance in bunching onions and related crops.

Round 2

Reviewer 3 Report

Comments and Suggestions for Authors

can be accepted 

Reviewer 4 Report

Comments and Suggestions for Authors

no comments.