# Supplementary material for: Metabolite Profiling and Association Analysis of Leaf Tipburn in Heat-Tolerant Bunching Onion Varieties
Source: Plants (Basel). 2025 Jan 11;14(2):187. doi: 10.3390/plants14020187 (PMC11768682; doi:10.3390/plants14020187)
Supplement: Supplementary file 1 [file plants-14-00187-s001.zip › LT_Figure S.pdf]

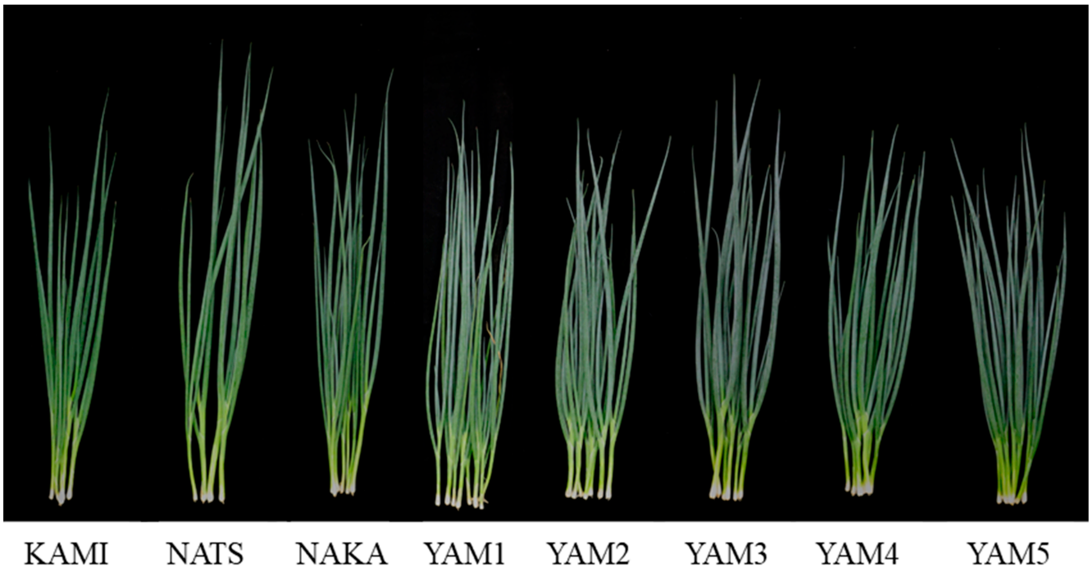

Figure S1. Plants from samples sown in May and harvested in July.

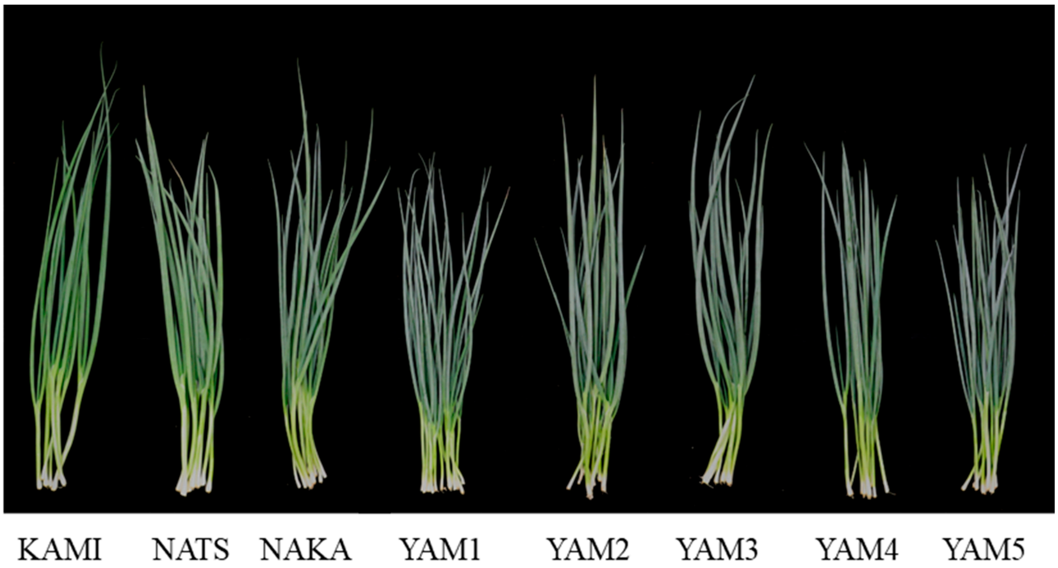

Figure S2. Plants from samples sown in May and harvested in August.

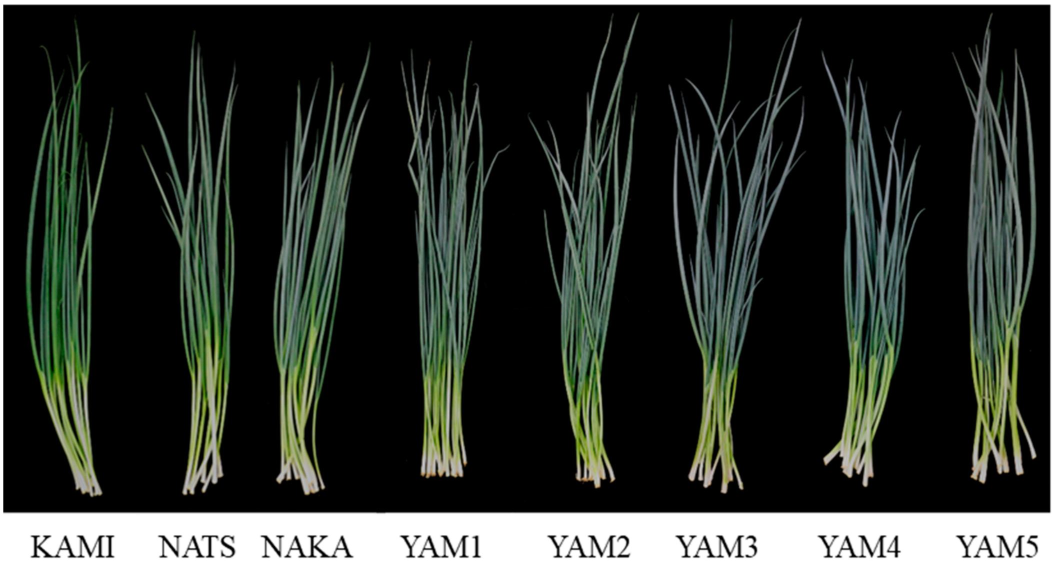

Figure S3. Plants from samples sown in May and harvested in September

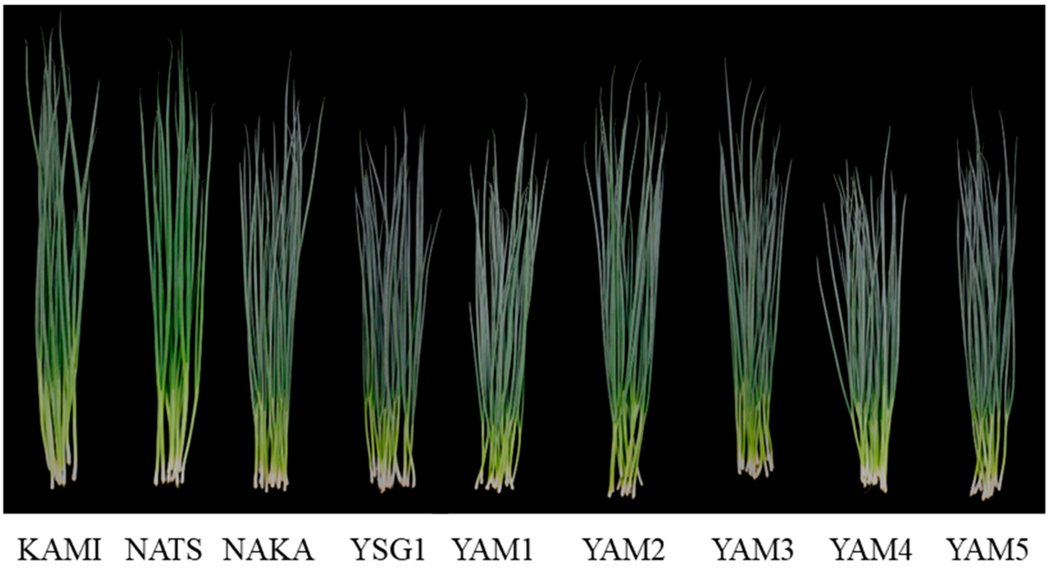

Figure S4. Plants from samples sown in June and harvested in August.

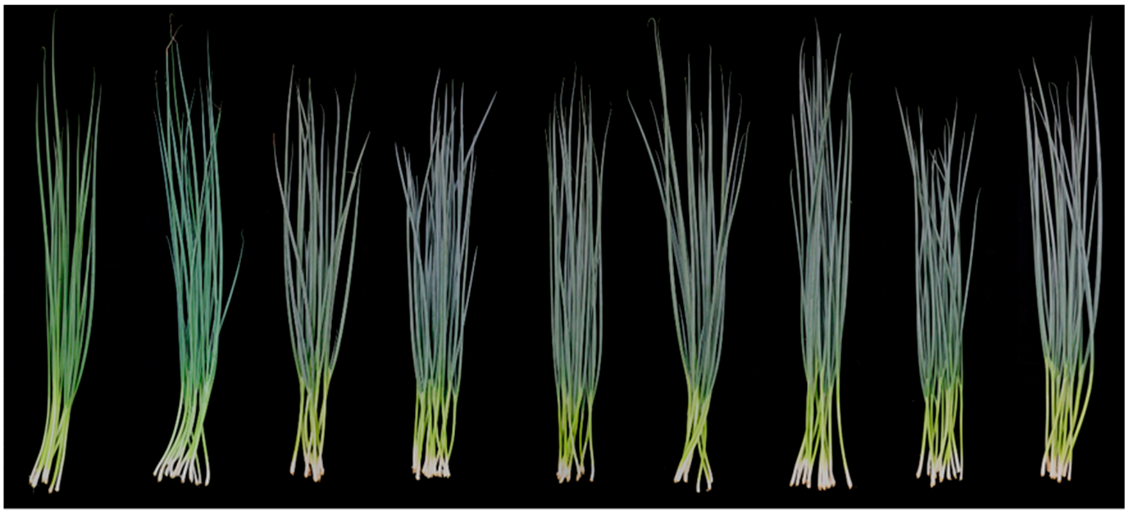

KAMI NATS NAKA YSG1 YAM1 YAM2 YAM3 YAM4 YAM5

Figure S5. Plants from samples sown in June and harvested in September.

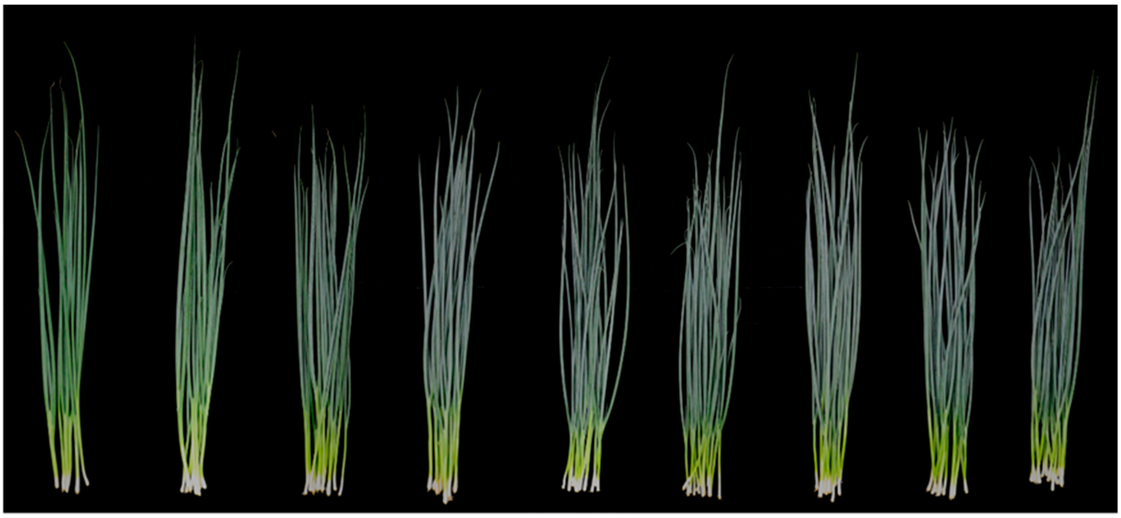

KAMI NATS NAKA YSG1 YAM1 YAM2 YAM3 YAM4 YAM5

Figure S6. Plants from samples sown in July and harvested in September.

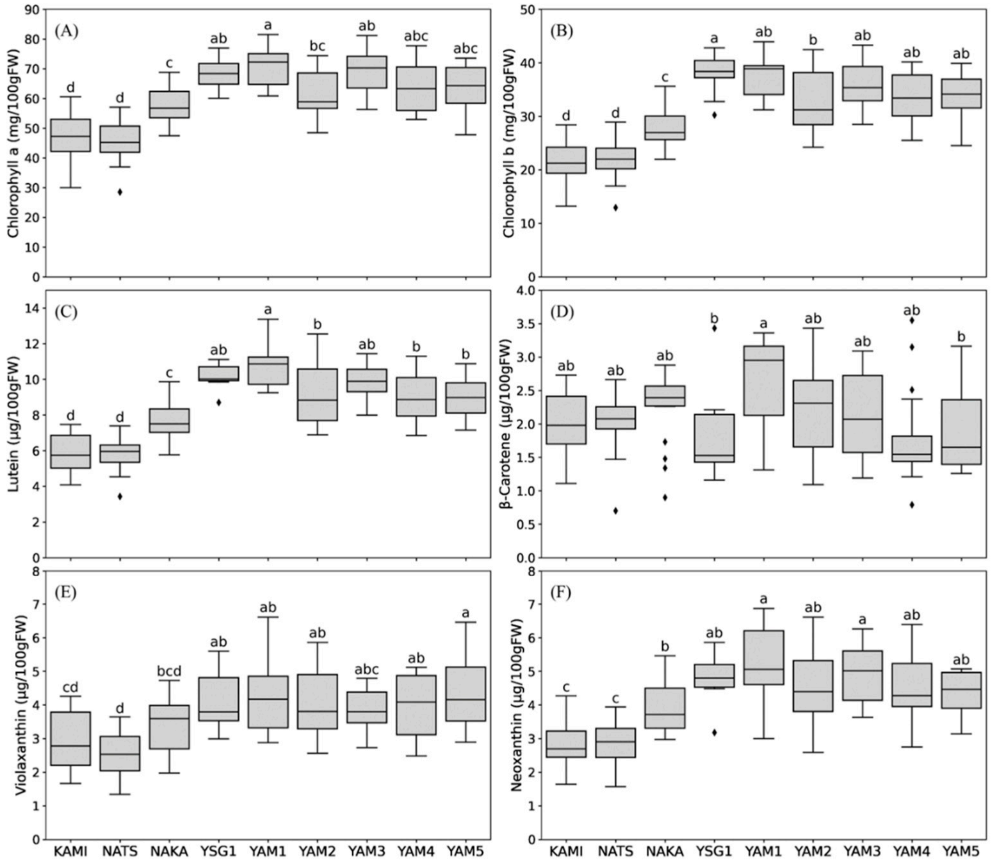

Figure S7. Boxplot of pigment compounds, including chlorophyll *a* (A), chlorophyll *b* (B), lutein (C), *β*-carotene (D), violaxanthin (E), and neoxanthin (F), across varieties and lines under all growing conditions. Different letters denote significant differences at $p < 0.05$.

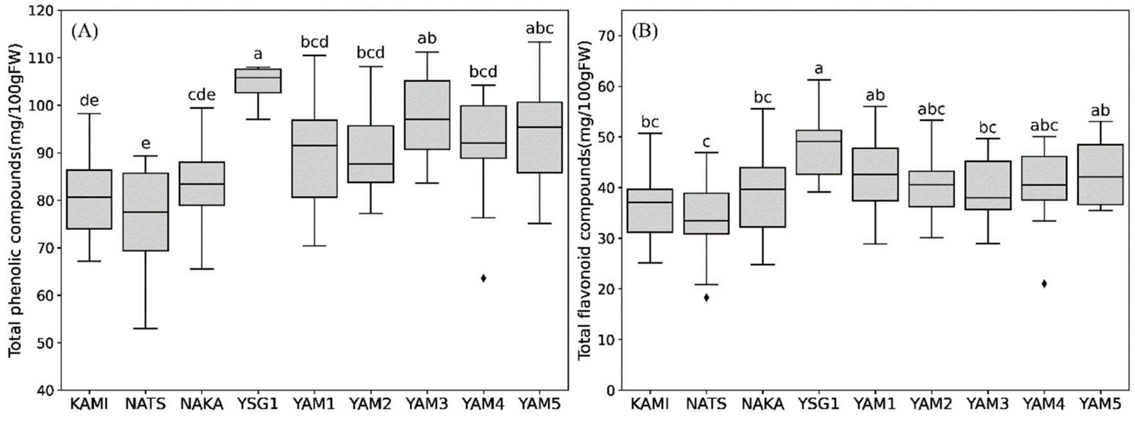

Figure S8. Boxplot of functional components, including total phenolic compounds (A) and total flavonoid compounds (B), across varieties and lines under all growing conditions.

Different letters denote significant differences at $p < 0.05$.

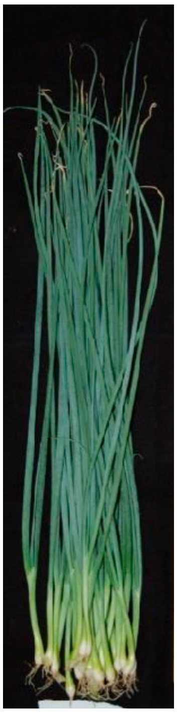

Figure S9. The plant of Asagi-Kujo harvested on August 18, 2017, used as a training image for supervised learning analysis.
